# Quadricuspid Aortic Valve: A Case Report of an Asymptomatic Middle-Aged Man

**DOI:** 10.7759/cureus.45262

**Published:** 2023-09-14

**Authors:** Abdulrahman Al-Qaysi, Naser Sayeh, Zainab Al-Qaysi, Yussef Al Ghoul, Essra Elhouni

**Affiliations:** 1 General Practice, High Street Medical Centre, Dungarvan, IRL; 2 Cardiology, University of British Columbia, Vancouver, CAN; 3 Internal Medicine/Mountain Vista Medical Centre, Avalon University School of Medicine, Mesa, USA; 4 Pulmonary Disease and Critical Care Medicine, Virginia Commonwealth University, Richmond, USA; 5 Pediatrics, Zawia University, Zawia, LBY

**Keywords:** asymptomatic, echocardiography, middle age, incidental finding, quadricuspid aortic valve

## Abstract

A quadricuspid aortic valve (QAV) is a rare congenital anomaly characterized by the presence of four leaflets in the aortic valve. We are reporting a case of a 59-year-old male who presented to the emergency department with non-cardiac chest pain. The discovery of QAV during the evaluation highlights the importance of considering cardiac causes, even in cases where the presenting symptoms may not appear directly related to the heart.

## Introduction

A quadricuspid aortic valve (QAV) is indeed a rare anomaly, with an incidence of less than 0.05% [1], making it quite uncommon. Transthoracic echocardiography is a commonly used non-invasive imaging technique, and for more accurate and detailed visualization, transesophageal echocardiography is often employed [2-4]. Other less frequently used methods of imaging such as cardiac computed tomography (CT) and cardiac magnetic resonance imaging (MRI) may provide more distinct valve morphology [4], however, some experts do not recommend the utilization of those tests in clinical practice [5]. One important aspect to note is that QAV may be associated with other aortic anomalies, such as aortic regurgitation, and additional cardiac abnormalities [6,7]. Moreover, QAV can be asymptomatic, and its presentation may be incidental [8], as in our patient's case. Although certain information may appear repetitive, our aim is to offer supplementary insights to address the existing gaps in the literature and to increase awareness among healthcare professionals.

## Case presentation

A 59-year-old male with a history of asthma and no previous history of cardiac diseases presented to the emergency department with chest pain. The chest pain was described as dull, occurring at rest, and localized in the lower chest and upper abdomen. There were no associated symptoms such as shortness of breath or nausea. The patient did not have a history of syncope or palpitations. The initial examination of the patient revealed vital signs within normal limits, including blood pressure (131/84 mmHg), heart rate (65 beats per minute), and oxygen saturation (99% on room air). Cardiac examination was unremarkable, with normal heart sounds, and no murmurs or rubs. The chest was clear on auscultation. Laboratory tests, including complete blood count, electrolytes, and kidney function, were all normal. The lipid profile results were as follows: total cholesterol was 5.81 mmol/L (reference range: 3.5-5.2 mmol/L), low-density lipoprotein (LDL) was 2.96 mmol/L (reference range: <3.0 mmol/L), high-density lipoprotein (HDL) was 1.05 mmol/L (reference range: >1.0 mmol/L), and triglycerides was 2.45 mmol/L (reference range: <1.7 mmol/L). Troponin levels were negative. Given the patient's history of dyslipidemia and family history of ischemic heart disease (IHD), the patient was referred to cardiology for further evaluation. The cardiology report showed an unremarkable electrocardiogram (ECG) with sinus rhythm and incomplete right bundle branch block. A stress test was performed, which came back negative at a good workload, suggesting no inducible myocardial ischemia. A 24-hour Holter monitor depicted normal sinus rhythm overall, with very rare premature ventricular contractions (PVCs) and premature atrial contractions (PACs).

**Figure 1:**
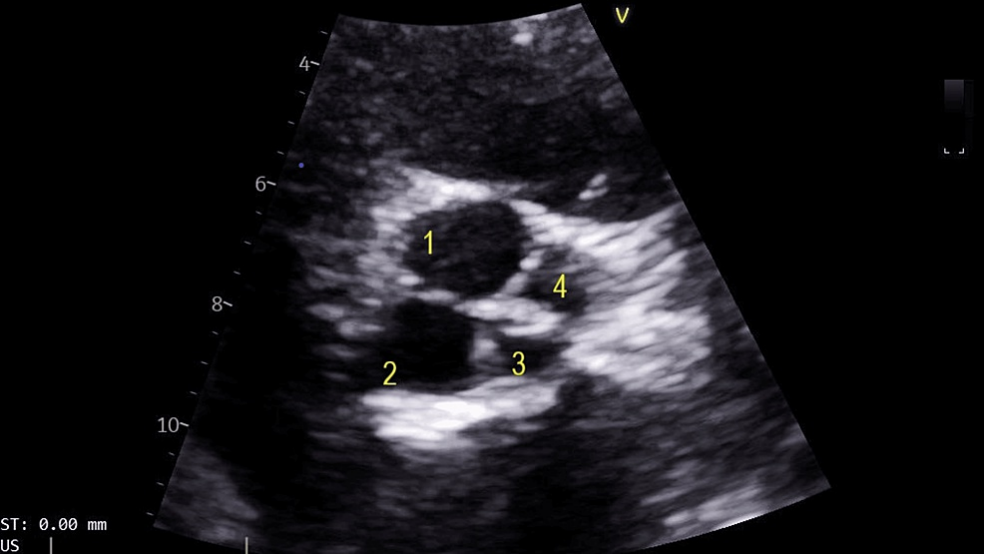
Transthoracic echocardiogram, diastolic view

**Figure 2:**
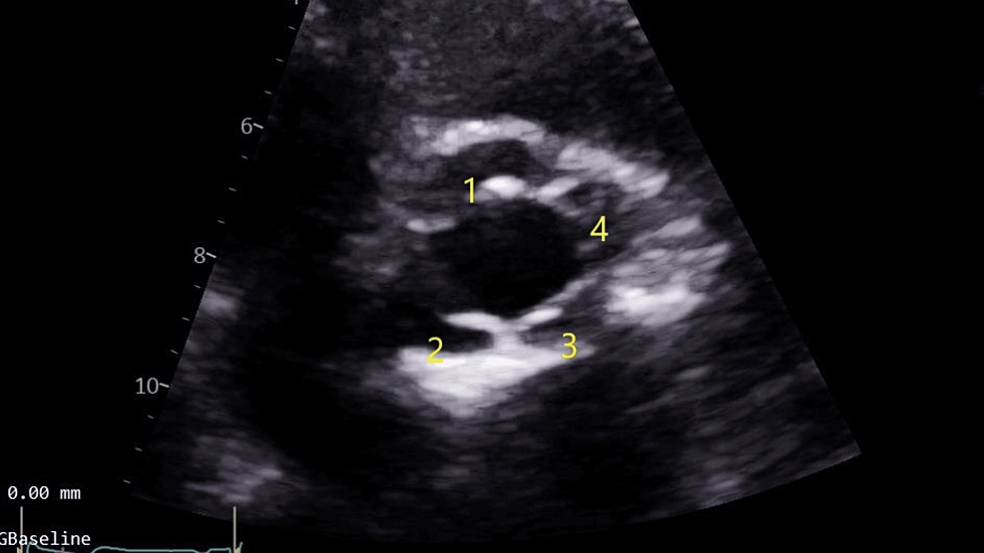
Transthoracic echocardiogram, systolic view

**Figure 3:**
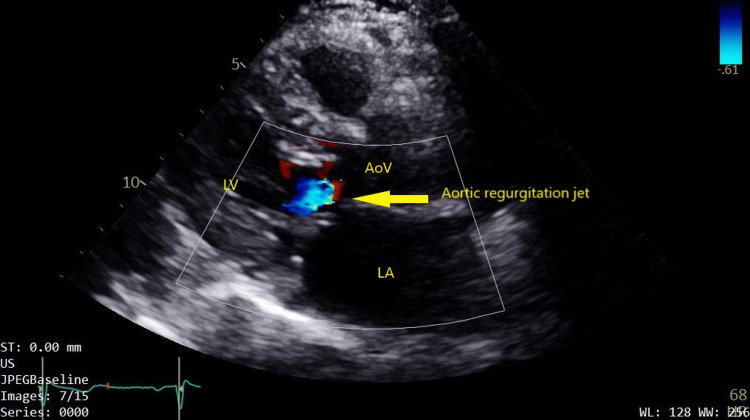
Aortic insufficiency AoV: aortic valve. LA: left atrium. LV: left ventricle

Subsequent transthoracic echocardiography was performed and revealed normal left ventricular size and systolic function, with an ejection fraction of 64%. The right ventricle and left atrium were also normal in size and function. Both the mitral and tricuspid valves showed normal structure and function. However, the aortic valve was found to be quadricuspid (Figures 1, 2; Video 1) with mild aortic regurgitation (Figure 3). There was aortic valve sclerosis too but without leaflet restriction. The proximal aorta was mildly dilated, measuring 38 mm. The patient has been followed up annually with echocardiography, and there has been no significant change in the results.

**Video 1:** Transthoracic echocardiogram shows four cusps in the aortic valve

## Discussion

In this case study, the quadricuspid aortic valve was discovered incidentally, and the patient remained asymptomatic, not requiring any treatment for many years after the diagnosis. Furthermore, it is worth mentioning that aside from the aortic root dilation, there were no other associated anomalies in this patient. Our goal in presenting this case is to offer fresh perspectives that contribute to the existing knowledge in the literature.

Quadricuspid aortic valve (QAV) is a rare congenital heart anomaly. The exact mechanism behind the development of QAV remains unknown, but it is believed to be related to abnormal septation during the early stages of heart development, potentially triggered by inflammatory episodes [5]. Hurwitz and Roberts described several anatomical subtypes of QAV [9]; however, there is currently no evidence suggesting that these subtypes have a significant influence on the clinical outcome or the severity of the condition [10].

The clinical presentation of QAV can vary depending on the age of diagnosis and the valve morphology [8]. Younger patients with QAV are often asymptomatic, however, as patients get older, they may develop symptoms related to aortic regurgitation or aortic stenosis [8]. Other sources have also indicated that the majority of individuals with QAV typically develop aortic insufficiency in their fifth or sixth decade, with approximately 20% of them requiring surgical intervention [4].

Due to the association of QAV with additional cardiac anomalies, it is recommended that patients with a diagnosed QAV valve undergo regular cardiac screening. This helps identify any coexisting heart abnormalities, which can have implications for the overall management and treatment of the condition [6]. However, there is a limited availability of data to provide precise follow-up recommendations. Treatment options, such as surgical intervention, may be considered based on the severity of the valve abnormality and associated cardiac conditions [6,11].

There have been some studies suggesting an increased risk of infective endocarditis in patients with QAV [5]. It has been thought that the abnormal valve morphology may contribute to altered blood flow patterns, turbulence, and potential areas of endothelial damage, which can increase the risk of bacterial attachment and subsequent infection [12]. The decision to provide antibiotic prophylaxis for dental procedures or other invasive procedures in patients with QAV is a topic of ongoing debate among medical professionals. Nonetheless, the American Heart Association (AHA) guideline update in 2008 does not recommend routine antibiotic prophylaxis for QAV unless there is evidence of active infection [13].

## Conclusions

The quadricuspid aortic valve is a rare congenital anomaly that can be found incidentally. This case highlights the possible manifestations of QAV, the diagnostic methods utilized, and the crucial role of routine cardiology follow-up. 

## References

[1] Feldman BJ, Khandheria BK, Warnes CA, Seward JB, Taylor CL, Tajik AJ (1990). Incidence, description and functional assessment of isolated quadricuspid aortic valves. Am J Cardiol.

[2] Godefroid O, Colles P, Vercauteren S, Louagie Y, Marchandise B (2006). Quadricuspid aortic valve: a rare etiology of aortic regurgitation. Eur J Echocardiogr.

[3] Nishimura RA, Otto CM, Bonow RO (2014). 2014 AHA/ACC Guideline for the Management of Patients With Valvular Heart Disease: executive summary: a report of the American College of Cardiology/American Heart Association Task Force on Practice Guidelines. Circulation.

[4] Yuan SM (2016). Quadricuspid aortic valve: a comprehensive review. Braz J Cardiovasc Surg.

[5] Savino K, Quintavalle E, Ambrosio G (2015). Quadricuspid aortic valve: a case report and review of the literature. J Cardiovasc Echogr.

[6] Tsang MY, Abudiab MM, Ammash NM, Naqvi TZ, Edwards WD, Nkomo VT, Pellikka PA (2016). Quadricuspid aortic valve: characteristics, associated structural cardiovascular abnormalities, and clinical outcomes. Circulation.

[7] George BA, O'Hayre TA, Schussler JM (2013). Association between congenitally quadricuspid aortic valve and mitral valve prolapse. Proc (Bayl Univ Med Cent).

[8] Khatun N, Kaliounji A, Alkoutami SS, Francois J, John S (2023). Quadricuspid aortic valve: an incidental finding in an elderly man. Cureus.

[9] Hurwitz LE, Roberts WC (1973). Quadricuspid semilunar valve. Am J Cardiol.

[10] Nakamura Y, Taniguchi I, Saiki M, Morimoto K, Yamaga T (2001). Quadricuspid aortic valve associated with aortic stenosis and regurgitation. Jpn J Thorac Cardiovasc Surg.

[11] Mecozzi G, Pratali S, Milano A, Nardi C, Bortolotti U (2003). Severe quadricuspid aortic valve stenosis after mediastinal irradiation. J Thorac Cardiovasc Surg.

[12] Dajani AS, Taubert KA, Wilson W (1997). Prevention of bacterial endocarditis. Recommendations by the American Heart Association. Circulation.

[13] Bonow RO, Carabello BA, Chatterjee K (2008). 2008 Focused update incorporated into the ACC/AHA 2006 guidelines for the management of patients with valvular heart disease: a report of the American College of Cardiology/American Heart Association Task Force on Practice Guidelines (Writing Committee to Revise the 1998 Guidelines for the Management of Patients With Valvular Heart Disease): endorsed by the Society of Cardiovascular Anesthesiologists, Society for Cardiovascular Angiography and Interventions, and Society of Thoracic Surgeons. Circulation.

